# The CHALO! Study Results of a Randomized Controlled Trial to Reduce Risk of Childhood Dental Caries and Obesity

**DOI:** 10.3390/ijerph23070837

**Published:** 2026-06-25

**Authors:** Arundhati Debnath, Karen Bonuck, Qi Gao, Usha Ramachandran, Sunanda Gaur, Christie L. Custodio-Lumsden, Dorota T. Kopycka-Kedzierawski, Mimi Kim, Alison Karasz

**Affiliations:** 1 Family Medicine and Community Health, UMass Chan Medical School, 55 N Lake Avenue, Worcester, MA 01655, USA; alison.karasz@umassmed.edu; 2 Department of Family and Social Medicine, Albert Einstein College of Medicine, 1300 Morris Park Avenue, New York, NY 10461, USA; karen.bonuck@einsteinmed.edu; 3 Department of Epidemiology & Population Health, Albert Einstein College of Medicine, 1300 Morris Park Avenue, New York, NY 10461, USA; qi.gao@einsteinmed.edu (Q.G.); mimi.kim@einsteinmed.edu (M.K.); 4 Department of Pediatrics, Robert Wood Johnson Medical School, 125 Paterson St., New Brunswick, NJ 08901, USAgaursu@rwjms.rutgers.edu (S.G.); 5 College of Dental Medicine, Columbia University, 622 W 168th St., New York, NY 10032, USA; clc2123@cumc.columbia.edu; 6 Eastman Institute for Oral Health, University of Rochester Medical Center, 625 Elmwood Avenue, Rochester, NY 14642, USA; dorota_kopyckakedzierawski@urmc.rochester.edu

**Keywords:** dental caries, obesity, oral health, South Asian, children, common risk factor approach

## Abstract

**Highlights:**

**Public health relevance—How does this work relate to a public health issue?**

- Childhood obesity and oral caries are linked to metabolic diseases in adulthood, a major public health concern.
- This intervention employed a common risk factor approach to reduce the risk of obesity and oral caries among high-risk, low-income South Asian immigrant children in the US.

**Public health significance—Why is this work of significance to public health?**

- This intervention was successful in improving child diet and health-promoting behaviors, such as increased dental visits, and also successful in reducing maternal feeding practices that increase caries and obesity risk.
- Findings suggest engaging other decision-making family members could lead to more robust changes in weight and oral caries outcomes.

**Public health implications—What are the key implications or messages for practitioners, policy makers and/or researchers in public health?**

- Social context and social constraints influence mothers’ ability to make child feeding decisions and change their children’s feeding behaviors.
- Future research should aim to influence maternal social networks to promote stronger and more sustained child feeding behavior change.

**Abstract:**

(1) Background: Obesity and dental caries disproportionately affect low-income South Asian (SA) immigrant children in the US. This CHALO! study aimed to reduce the risk of obesity and oral health risk in young SA children in the US. (2) Methods: CHALO! is a randomized controlled trial. A total of 350 low-income Bangladeshi mothers of 6-month-old children were recruited and randomized to intervention or control. Intervention participants received six home visits and six phone calls from trained community health workers who delivered health education and support. The primary outcome was frequency of combined bottle/sippy cup use over 18 months measured via self-report. Secondary outcomes included sugar consumption, maternal feeding practices, oral hygiene practices, and dental utilization measured via self-report. Secondary clinical outcomes included the presence of dental caries at follow-up (12 months post baseline) assessed through intra-oral camera, and obesity risk, measured as weight gain velocity, at each 6-month period. (3) Results: Bottle/sippy-cup use increased less in the intervention group (Poisson rate ratio = 0.36, 95% CI: 0.34–0.39, *p* < 0.0001) vs. controls (Poisson rate ratio = 0.58, 95% CI: 0.56–0.61), and while consistent results were noted in sugar consumption, oral hygiene practices, dental visits, and other secondary outcomes, no difference was found in caries prevalence or weight gain velocity. (4) Conclusions: The intervention improved self-reported bottle use and child diet in the intervention group. There were no significant changes in caries prevalence or weight gain velocity. Social context, particularly social networks, may act as a barrier to adopting new healthy behaviors, impacting changes in caries and obesity outcomes.

## 1. Introduction

Over the past several generations, societies around the world have passed through a historical change in food environment known as the nutrition transition. Pre-transition environments include highly nutritional foods and are often characterized by calorie deficits, while post-transition environments include low nutrition, high-calorie diets and a calorie surplus [1]. The result is an epidemic of nutrition-related diseases, particularly caries and obesity-related conditions, with lower-income and immigrant communities particularly affected [2,3]. Childhood obesity and dental caries are among the two most prevalent child health disparities [4,5,6,7,8,9,10].

In the United States (US), both childhood obesity and caries disproportionately impact children from ethnic minorities and low socioeconomic status (SES) communities [11,12]. The Common Risk Factor approach to reducing oral health disparities focuses on addressing the determinants of dental caries that are shared with other diseases of modern life, including obesity and metabolic disease [13]. Approaching common behavioral risk factors, especially related to dietary sugar and refined carbohydrates, simultaneously, increases the cost effectiveness of interventions designed to address these diseases [14], but has been little used in child health interventions. This study tested a common risk factor approach to childhood obesity and dental caries among young South Asian immigrant children.

South Asians (SAs) are among the fastest growing immigrant groups in the US, with a population of nearly 5.4 million. Several decades ago, the South Asian diaspora included mainly professionals, who acculturated quickly and dispersed to affluent suburbs. More recently, working-class immigrants from Pakistan and Bangladesh have arrived in large numbers. These cluster in urban areas characterized by poverty, cultural isolation, and unmet service needs [4,12]. Low-income South Asian children are at high risk for obesity [15,16]. They typically demonstrate a risky pattern of early growth: low birthweights followed by steep weight gain in infancy and early childhood [17]. South Asian immigrant children are also at comparatively higher risk for early childhood caries (ECC) [11,18,19], even adjusting for SES [20].

Several factors, both common and unique, play a role in child oral and obesity risk in South Asian (SA) communities. A key common risk factor is child diet. Consumption of sweetened beverages and sweet/salty snacks has been associated with childhood and adult obesity [21,22] and ECC [23,24]. Research shows that SA child diets are heavy in sweets and sweetened drinks, compared to other groups [25,26,27,28]. Another common risk factor involves child feeding practices [29]. Bottle (and sippy cup) use beyond 12–15 months increases the risk of both obesity [30,31,32] and ECC [33,34], and is more common in SA children than in other groups [35,36,37]. Other common risky feeding behaviors include the frequent use of bottle additives in SA families [27,28,38]. An indulgent feeding style, in which children are allowed to choose what they will eat, is often associated with high sweet consumption [39].

At the social level, another common risk factor playing a role in both child oral health and obesity risk is the disempowerment of SA mothers. In SA families, husbands and elders play a major role in child-rearing decision-making, especially in relation to feeding [29]. Family elders may be highly critical of mothers who try to reduce children’s consumption of risky foods.

Besides these common risk factors for obesity and dental caries, there are unique risk factors for ECC. In SA families, primary teeth may be viewed as dispensable and families may seek dental care only when the child is experiencing pain or visible decay [39]. Poor oral hygiene practices and low dental utilization are additional risk factors for ECC [40]. Guidelines recommend that children from low-income immigrant families should visit a dentist every six months [41,42]; however, only 1.5% of 12-month-olds visit a dentist each year [43], and these visits are lowest amongst low-income families [44].

Unique risk factors for obesity include pressured feeding, in which parents control feeding and children are pressured/forced to eat. Another unique risk factor for obesity is sedentary behavior [45,46,47]. SA children and adults are more sedentary than other groups [48,49]. Sedentary behaviors among South Asians are associated with a higher risk of developing cardiovascular and metabolic disorders [50].

A small number of child health interventions focusing on obesity in SA children have been conducted in South Asia [51,52], the UK [53,54,55,56,57], Australia [58] and Canada [59]. By contrast, little intervention research has been conducted on child oral health and obesity in US South Asian communities.

We employed the common risk factor approach in the design of the CHALO! trial [60,61]. The Child Health Action to Lower Oral health and obesity risk (CHALO!—from a Hindi/Bengali word meaning “Let’s go!”) was a culturally adapted home visiting intervention for young South Asian immigrant children in New York City. In developing the study design, we sought to replicate the strengths of several large-scale, well-designed child obesity trials reported in the literature [62,63]. These included home visits, which have been widely recommended for child health interventions [62,63,64,65,66], as well as intensive intervention schedules and culturally adapted materials. We replicated some of these features, adapting session plans and educational materials based on culture-specific risks, attitudes, and values [29].

The primary aim of the CHALO! study was to reduce risk behaviors associated with dental caries and obesity at 18 months. We hypothesized that the intervention group would demonstrate reduced risk compared to the control group—specifically, less bottle/sippy cup use.

The secondary aim included improved maternal feeding behaviors, child diet, oral hygiene and dental utilization, physical activity and reduced screen time. We hypothesized that the intervention group would demonstrate greater improvements in these behaviors compared to the control group. Secondary clinical aims included reduced incidence of caries and weight gain velocity at 18 months. We hypothesized reduced caries incidence and reduced weight gain velocity in the intervention vs. control group.

## 2. Materials and Methods

### 2.1. Study Design

The CHALO! study is a two-arm randomized controlled trial.

### 2.2. Participants, Sample Size and Recruitment

CHALO was targeted in low-income South Asian families in New York City. Between 2017 and 2020, 350 mothers of children aged 4–6 months old were enrolled.

The CHALO! trial was powered to detect treatment group differences in bottles + sippy cups (combined) from 12 to 18 months. Based on our previous bottle-weaning trial results [67], we estimated a difference in effect size (i.e., combined number of bottles + sippy cups/24 h) of 0.7. This equated to a small–moderate standardized effect size (Cohen’s d) of 0.32. Considering a 20% attrition rate, to detect a difference of 0.5 in the decline rate of bottle and sippy cup use, with 80% power at alpha = 0.05, the sample size was calculated to be *n* = 377 total with 189 mother–child dyads enrolled per arm.

Participants were identified via several methods. Some were recruited from pediatric practices in South Asian neighborhoods in the Bronx, Queens, and New Jersey. Other participants were recruited from announcements, flyers, and other outreach efforts at Sapna NYC. Inclusion criteria included: (a) aged six months (+/− 30 days) at time of the baseline interview; (b) child enrolled in either the Children’s Medicaid or Children’s Health Insurance program (CHIP), a publicly funded program for lower-income families not eligible for Medicaid; (c) mother’s birthplace in India, Pakistan, or Bangladesh; (d) mother fluent in standard Bengali or Hindi/Urdu; (e) mother is index child’s primary caretaker.

### 2.3. Randomization and Blinding

Participants were randomized 1:1 to intervention or control groups. The study statistician computer randomized the participants in advance, following which sealed envelopes were prepared with the random assignment. Following informed consent and a baseline interview, interviewers opened sealed envelopes and informed participants of their group assignment.

Data collectors were blinded to group assignments and were different from staff who delivered the intervention.

### 2.4. Data Collection

**Figure 1:**
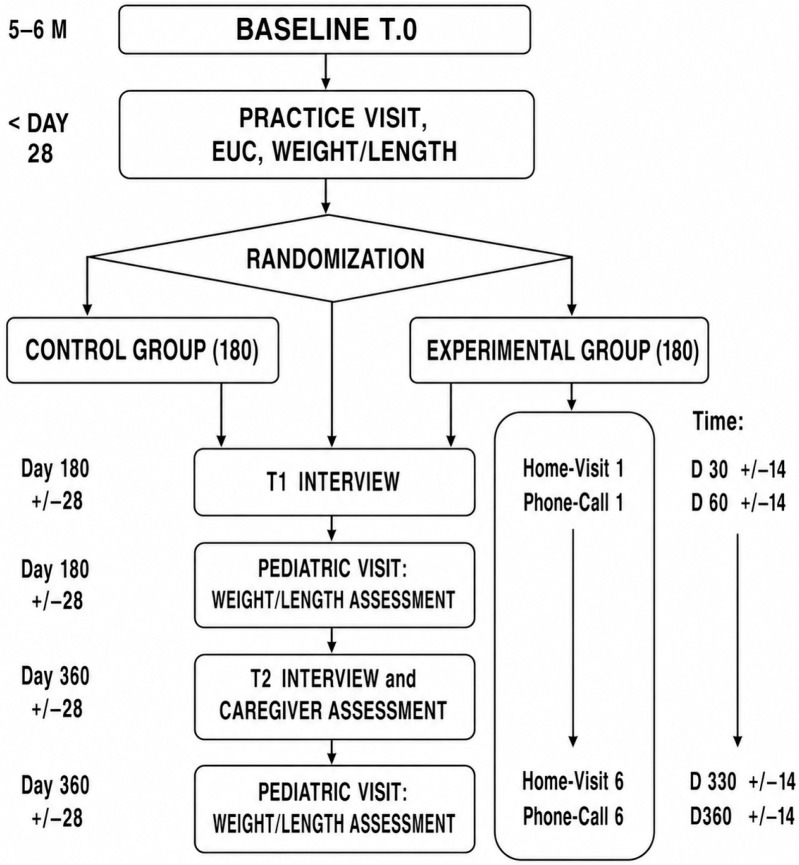
Flowchart.

Data collection (see Figure 1) was conducted between December 2017 and December 2021. Before February 2020, research assessments were conducted by bilingual Bengali/Urdu research assistants in the home. After February 2020, visits were conducted by phone or teleconference. Data collection included questionnaires (see Table 1 for a full list), anthropometric data, and dental caries data. Data collection on harm or unintended effects were conducted non-systematically; research staff documented any spontaneous reports of adverse events or distress volunteered by participants during study interviews.

### 2.5. Primary Outcome

The primary outcome of the RCT was the frequency of combined sippy cups and bottle use (count/week). Research has shown that valved sippy cups function similar to bottles and are linked with both ECC [73] and obesity [74].

### 2.6. Secondary Outcomes

Dietary: Secondary outcomes measured included child diet outcomes (e.g., sweet foods and drinks, snacks, bottle additives) and food delivery systems at nap or bedtimes (bottles and valved sippy cups). Two measures were used to assess dietary outcomes. The first was a culturally tailored, web-based, 24 h recall instrument called MySmileBuddy (MSB) [75]; the second was an adapted version of the Child Food Frequency Questionnaire (FFQ) used to identify frequency of foods consumed [68]. Technical problems with the online interface interfered with the implementation of the MSB. Consequently, only FFQ outcomes are reported [68].

Oral hygiene: Oral hygiene practices and dental utilization were measured by the Oral Hygiene and Dental utilization questionnaire (OHDU) [69]. This instrument assesses the frequency of toothbrushing and whether the child has ever visited a dentist.

Maternal Feeding: Maternal feeding behaviors and attitudes were measured by an adapted version of the Infant Feeding Styles Questionnaire (IFSQ) [70]. The measure assesses feeding beliefs linked to risky feeding practices, such as the belief that small children do not know when they are hungry or full, and maternal feeding behaviors such as pressured and indulgent feeding. The measure was adapted for use with SA mothers.

Physical activity: Physical activity was measured by the Early Years Physical Activity Questionnaire (EYPAQ). Items assessed the duration, intensity and frequency of physical activities over a 7-day period.

Screen time: Screen time was measured by an adapted Screen time questionnaire. This focused on the amount of time children spent using screens.

Caries Outcomes: The caries outcome was assessed by an oral exam using intra-oral dental cameras [76,77]. Intra-oral cameras are a valid [78,79,80,81] and acceptable [82] means of assessing ECC. In the present study, RAs attempted to capture up to six images: upper and lower front teeth, upper right and upper left, and the lower right and lower left biting surfaces of back teeth—depending upon the number of primary teeth present in the mouth. The study’s pediatric dentist determined base images and visible dental caries (yes/no).

### 2.7. Anthropometric Measures

Anthropometric measures (weight gain velocity): Calibrated research quality scales were used to assess weight by pediatric practice staff members. Before March 2020, these in-person techniques were used. After this date, we used weight data from a combination of sources. These included data from pediatric visits. Weight was typically recorded on cards given to the mother at these visits. In some cases, mothers sent the research team an image of the weight card. In other cases, the mother self-reported child weight, reading from the card. Weight gain velocity was calculated for each 6-month period.

### 2.8. Mediator Variables

We hypothesized that maternal feeding styles, beliefs, and practices, as measured by the IFSQ [70], would mediate the impact of the intervention on the primary outcome of combined bottle and/or sippy cup use.

### 2.9. Moderator Variable

An important variable in behavioral interventions is the quality of the relationship between the interventionist and participant [83]. To measure relationship quality between the CHW and participant, we used the Working Alliance Inventory (WAI) [72]. The measure includes items such as “___ and I are working towards mutually agreed upon goals”. It was adapted for the current study context and was administered at T2 (12 months post baseline). We hypothesized that stronger relationships, as measured by the WAI, would be linked to an improvement in our primary outcome.

### 2.10. Experimental Group Intervention

The CHALO intervention was designed to provide health education on childhood obesity and oral health risk prevention, along with goal setting, social support and navigation to a dental visit. The protocol consisted of six home visits when the child was 6, 8, 10, 12, 14, and 16 months old. The 90 min visit included rapport building, homework review, an educational component with a flip chart and handouts, and a goal setting component (see Table 2 for a description of the study sessions). Core goals for the intervention included: reduced sugary drinks and foods, reducing/stopping bottle feeding, reducing force/pressure feeding, reduced screen time, toothbrushing 2×/day, and at least one visit to the dentist during the project year. Flip charts with educational messages adapted to South Asian culture, family life, and nutritional practices were created, translated, and pretested in three focus groups before the commencement of the trial. Educational messages focused on topics like stopping breast feeding and bottle feeding, the impact of naptime and bedtime bottle feeding, increased consumption of fruits, vegetables and solid food, reducing consumption of milk, sweetened beverages, juice, sweetened and salty snacks, encouraging self-feeding, increasing physical activity and active play, reducing screen time, oral health and caring for baby’s teeth.

In addition to providing education, CHWs worked with mothers on skill building. Skills included: recognizing infants’ hunger cues, preparing appropriate self-feeding foods, and techniques for teeth brushing. At each visit, the CHW encouraged the mother to set a concrete, manageable behavior change goal and a plan for achieving the goal. For example, if the goal was to decrease the number of bottles of milk consumed—a major caries risk—the mother created a plan to gradually replace the milk in her child’s bottle with water. In addition to the in-person visits, follow-up telephone support was also provided by CHWs at ages 7, 9, 11, 13, 15 and 17 months. Follow-up calls focused on addressing challenges to the mothers’ behavioral goals.

The original design of the study included family members during the 8- and 14-month visits. The goal was to involve family decision makers, e.g., husbands or mothers-in-law. Family visits were designed to empower the mother and support child health decision-making by assuring ‘buy in’ from household decision-makers. However, it proved difficult to engage these family members and the family component eventually dropped. In March of 2020, the pandemic effectively ended home visits for our study, and both intervention and data collection visits continued by phone.

The third component of the intervention included support for a dental visit. Because many dental practices in the area did not accept patients as young as 12 months of age or did not accept the insurance plans of many of the participants, the study team developed a list of vetted dentists in each neighborhood. Study staff, identifying as a parent, telephoned dental offices to inquire about the ages of the children and insurances accepted. Participants in the study received a copy of the list. Though the original goal was for the child to make two dental visits during the intervention period, as recommended by the AAPD [41], it turned out that public insurance plans would cover costs for only one visit. Consequently, the goal was changed to one visit during the intervention period.

**Figure 2:**
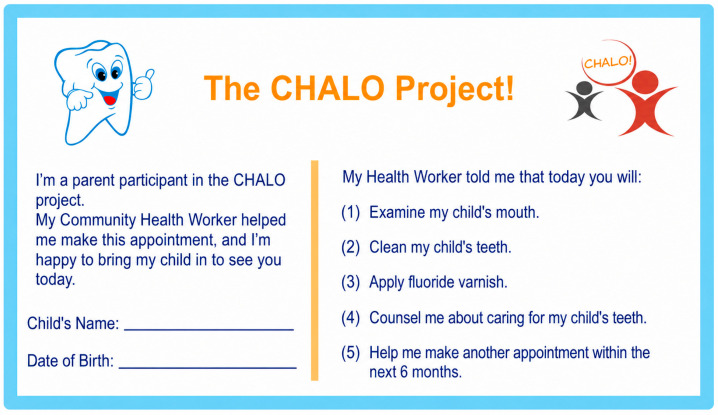
Doc talk card which participants showed to the dentist.

To encourage dental visits, mothers received guidance and support on making and attending office visits. In many cases, the patient navigator made the appointment for the mother. Car fare was provided for office visits that were not within walking distance. In preparation for their dental visits, mothers received information about the benefits of fluoride varnish and were encouraged to request a varnish application during their visits. Each intervention participant received a “doc talk” (see Figure 2) card to bring with her to the dentist. The card identified the mother as a CHALO! study participant and requested that the dentist examine the child’s teeth and apply a dental varnish.

### 2.11. Control Group Intervention

The control group received pamphlets with messages related to early childhood caries and obesity prevention, along with the same lists of local vetted dentists provided to the intervention group.

### 2.12. Analysis

#### 2.12.1. Descriptive Analyses

Mean and standard deviation or median and interquartile range (IQR) were reported for continuous variables, while frequency and percentage were reported for categorical variables. To compare the difference between treatment arms at baseline (T0), Student’s *T*-test or the Wilcoxon rank sum test were performed for continuous variables, while the Chi-square test or Fisher’s exact test were performed for categorical variables.

#### 2.12.2. Main Analyses

For the primary outcome, weekly frequency of combined bottle/sippy cup use, and for the secondary outcomes, which included weekly consumption of bottles with additives, vegetable servings, juice, sugary drinks, weekly bottle use before bed/naptime, and weekly frequency of teeth cleaning—data were collected at T0, T1 and T2. To assess the treatment effects on these outcomes, generalized linear mixed effects models (GLMMs) with random intercept terms (subject effect) were fit to the data. Depending on the distribution of the data, Poisson or negative binomial distributions were assumed and the log-link was used. For sweets and salty foods, and total unhealthy foods, the outcomes were categorized into 0 and tertiles (low, medium and high levels) and analyzed as a multinomial outcome with a cumulative logit link in the GLMM. All GLMMs include month, treatment indicator, and their interaction terms as the main effects, adjusted for baseline household income, mom’s education, years of mom residing in the US, heritage score, mainstream score, tension scale, economic decision, family size, freedom of movement, coercive control, and gender attitude scores. The interaction term estimated the rate ratio change in outcome attributable to the intervention.

To test the impact of the intervention on weight gain velocity, participants’ weight was measured multiple times at T0, T1 and T2 and similar linear mixed effects models were fit to the data, with month, treatment indicator and their interaction term. The interaction term estimated the difference in growth velocity between two treatment groups.

To examine the treatment effects for the secondary outcomes measured at T2 only, such as screen time and physical activity, linear regression models were used, with treatment as the main effect, adjusting for the same covariates listed above. As for the dentist visit (Yes/No) and caries, a logistic regression model was used to analyze this binary outcome.

To address missing data, sensitivity analysis was carried out. Missing baseline covariate data were handled using multiple imputation for the primary outcome—combined bottle/sippy cup usage. Twenty imputed datasets were generated using fully conditional specifications, incorporating all baseline covariates, treatment assignment, month, and outcome measures. Generalized linear mixed effects models were fit separately within each imputed dataset, including treatment, month, and their interaction as fixed effects. Parameter estimates were pooled across imputations. See Supplementary Table S3 for multiple imputation results.

#### 2.12.3. Mediation Analysis

To examine whether IFSQ score mediated the effect of the intervention on change in sippy cup and bottle use between T0 and T2, the SAS PROC CAUSALMED 9.4 (SAS Institute Inc., Cary, NC, USA) procedure was used. Change in sippy cup/bottle use was specified as the outcome variable, change in IFSQ score as the mediator, and the same covariates were adjusted for as in the above analyses, along with baseline sippy cup/bottle use, to estimate the percentage of the intervention effect mediated by IFSQ.

#### 2.12.4. Moderator Analysis

To test whether the relationship between the participant and CHW moderated the rate ratio of bottle feeding over time in the intervention group only, a generalized linear mixed effects model was fit to the data with Poisson distribution. Working Alliance mean scores were categorized into low, medium and high levels based on tertiles, and included in the model, together with month and their interaction terms. The model also adjusted for the same covariates as above. The interaction terms were used to evaluate the magnitude and significance of the moderating effects of the working alliance on rate of change in the outcomes.

An alpha level of 0.05 was considered statistically significant. All analyses were performed using SAS 9.4 (SAS Institute Inc., Cary, NC, USA).

#### 2.12.5. Interim Analysis

No interim analyses were planned or conducted for this trial, and no formal data-dependent stopping guidelines were established.

## 3. Results

The baseline sample consisted of *n* = 350 mother–child dyads. Of these, *n* = 176 were randomized to the intervention group and *n* = 174 were randomized to the control group (see Table 3 for participant demographic characteristics). A total of 44 participants were lost to follow-up, for a retention rate of 87.42%. See Supplementary Table S1 for sample sizes in analysis across different timepoints and Supplementary Table S2 for baseline comparison of intervention vs. control groups of outcome variables.

### 3.1. Primary Outcome

**Figure 3:**
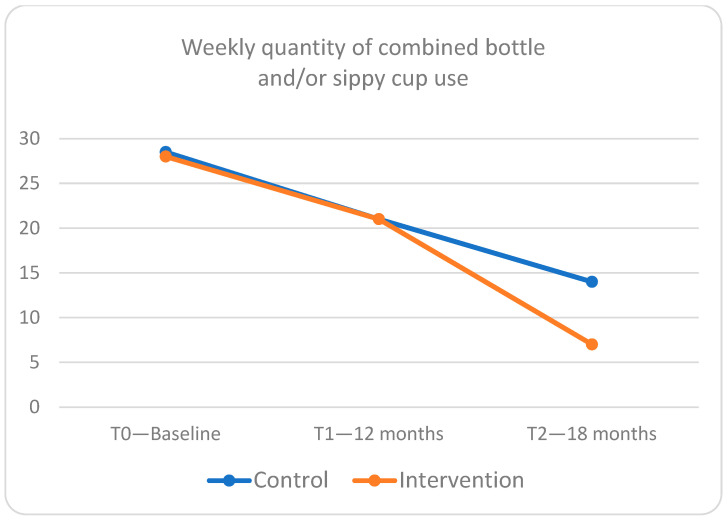
Weekly quantity of combined bottle and/or sippy cup use over time.

Self-reported sippy cup and bottle use declined over time in both the control and intervention groups, but the rate of decline was greater in the intervention group (see Figure 3). Specifically, the median counts per week of sippy cup and bottle use in the control and intervention groups were 28.5 (IQR 56) versus 28 (IQR 49), respectively, at T0 (*p* = 0.45); 21 (IQR 35) versus 21 (IQR 28) at T1 (*p* = 0.17), and 14 (IQR 28) versus 7 (IQR 21) at T2 (*p* = 0.11) (Table 4 and Figure 3). The median changes between T0 and T1 in sippy cup and bottle use in the control and intervention groups were −4 (IQR 21) and 0 (IQR = 28), respectively, (*p* = 0.23); the median change between T0 and T2 were −7 (IQR 28) and −21(IQR 29), respectively, (*p* = 0.02). When analyzing the repeated measures of the primary outcome in a combined analysis, the estimated annual decline in the rate of combined sippy cups and bottle use, as measured by the Poisson rate ratio, is 0.58 (95% CI: 0.56–0.61) in the control group and 0.36 (95% CI: 0.34–0.39) in the intervention group. That is, the frequency of sippy cups and bottle use was estimated to decline at a yearly rate of (1 − 0.58) 42% in the control group and (1 − 0.36) 74% in the intervention group, indicating a greater rate of decrease in sippy cup and bottle use over time in the intervention group after controlling for covariates (*p* < 0.0001 based on time x treatment interaction).

To address missing data for the primary outcome, sensitivity analysis was carried out. Multiple imputation was performed to address missing data. Results of the multiple imputation were consistent with those of the primary analysis.

### 3.2. Secondary Outcomes

We observed a broad pattern of significant treatment effects, including significant changes in child diet, maternal feeding styles, and oral hygiene practices. As expected, many risky dietary practices increased over the study duration; however, the rate of increase in these behaviors was greater in the control group than the intervention group. The control group showed significantly greater increases in the rate of fruit juice consumption (control group rate ratio = 4.66, 95% CI: 3.87–5.61, vs. intervention group rate ratio = 2.45, 95% CI: 1.92–3.12; *p* < 0.0001), sweet and salty snacks (control group odds ratio = 40.68, 95% CI: 22.90–72.24, vs. intervention group odds ratio = 19.9, 95% CI: 11.11–35.67; *p* = 0.05), and unhealthy foods (control group odds ratio = 50.62, 95% CI: 28.48–89.96, vs. intervention group odds ratio = 16.83, 95% CI: 9.67–29.3; *p* = 0.0023). For other secondary outcomes, we saw a greater decrease in the frequency of behaviors in the intervention group compared to the control group. These behaviors were nap/bedtime sippy cup and/or bottle use (intervention group rate ratio = 0.23, 95% CI: 0.17–0.33, vs. control group rate ratio = 0.62, 95% CI: 0.49–0.79; *p* < 0.0001), and the percentage of screen time use (*p* = 0.0244). The rate of increase in the consumption of fruits and vegetables was greater in the intervention group (rate ratio = 2.30, 95% CI: 2.16–2.46; *p* = 0.0002) vs. the control group (rate ratio = 1.94, 95% CI: 1.82–2.06). Dental visits percentage was also significantly higher in the intervention vs. the control group (37.25% vs. 3.8%, *p* < 0.0001).

We found no significant difference across the groups in the prevalence of caries and weight gain velocity (see Table 4 for results of primary and secondary outcome variables at T0, T1, and T2 for intervention and control groups).

#### 3.2.1. Mediation Analysis

A causal mediation analysis was performed to assess whether the significant intervention effect on change in sippy cup/bottle use between T0 and T2 was mediated by a corresponding change in IFSQ. The percentage of the intervention effect on change in sippy cup/bottle use that was mediated by change in IFSQ was less than 1% (0.99%; 95% CI: −15.6%, 17.4%; *p* = 0.91), indicating that the intervention’s effect on the change in the primary outcome was not mediated by the change in IFSQ (see Table 5).

#### 3.2.2. Moderator Analysis

Among participants in the intervention group, the quality of the CHW/participant relationship, as measured by the WAI, had a moderating effect on the rate of change in combined sippy cup and bottle use. Specifically, the estimated annual decline in the rate of combined sippy cups and bottle use, as measured by the Poisson rate ratio, was 0.46 (95% CI: 0.39–0.53) among subjects in the first tertile of WAI scores, corresponding to a 54% annual decline, 0.38 (95% CI: 0.35–0.42) in the second tertile, corresponding to a 62% annual decline (*p* = 0.045 compared to first tertile), and 0.28 (95% CI: 0.25–0.32) in the third tertile, corresponding to a 72% annual decline (*p* < 0.0001 compared to first tertile), suggesting that a stronger alliance between CHW and participant led to significantly better outcomes.

## 4. Discussion

South Asian immigrant children are highly vulnerable to nutrition-related diseases such as oral caries [11,18,19,20] and obesity [15,16]. Identifying effective interventions for this group should be a public health priority. The CHALO! intervention was designed to provide health education to reduce the risk of childhood obesity and early childhood caries by reducing risky behaviors, along with providing support and skill building, to mothers. The design of the trial resembled previous successful interventions [63,84,85,86,87,88,89]. Working with a trusted community partner, Sapna NYC, we made considerable effort to create a culturally tailored intervention.

The intervention was effective in reducing bottle/sippy cup feeding behaviors—the primary aim of the study. Results also show improved self-reported child diet and maternal feeding behaviors, including improvements in fruit/vegetable consumption, along with reduced servings of juice, sweetened beverages, sweet and salty snacks, and bottle additives. Self-reported oral hygiene practices, such as toothbrushing, increased more in the intervention group compared to controls. Despite the pandemic, we found that intervention participants were more than 10 times more likely to visit a dentist during the study period, compared to controls (37.25% vs. 3.8%).

We did not see any differences in two secondary clinical outcomes: visible oral caries and weight gain velocity. Though previous research using the intra-oral camera approach was found to be an effective method of assessing visible caries [78,79,90], the prevalence rate in this study was extremely low, suggesting that the procedure may not have captured existing caries. The fact that we did not find changes in child obesity risk, as measured by change in weight velocity, may have been affected by the fact that, due to the COVID-19 pandemic, child height/length and weight data were collected from pediatrician records for a large proportion of the sample. This may have introduced error into these measurements. Results from a recent systematic review analyzing 17 randomized controlled trials found that parent-based behavioral interventions had no effect on preventing obesity in children aged 24 months (±6 months), also suggesting that a longer follow-up period may be needed to observe changes [91]. The pandemic may have impacted the study in other ways as well. It is possible that mothers became less engaged in the intervention after in-person visits stopped. However, the impacts on self-reported outcomes, including dental visits, suggest that mothers remained engaged in the study throughout the pandemic period.

To sum up, it appears that CHALO! was effective in improving self-reported behavioral outcomes such as maternal feeding practices and child diet. Yet, despite its careful cultural adaptation and intensity, and despite high retention rates (87.42%), the intervention may not have succeeded in changing caries and weight outcomes.

There were limitations to the CHALO! study: (1) Due to the COVID-19 pandemic, changes were made to the data collection methods mid-study—this included a change in collection of children’s height/weight data from pediatricians’ records, and other data collection was performed over the phone instead of in-person. (2) Another limitation may have been the measurement of visible oral caries using intra-oral cameras. (3) There was a lack of blinding in intervention delivery which may have introduced potential bias. (4) The 18-month (T2) follow-up period of study may have been too short to evaluate actual changes in the rate of weight gain in participant children.

Health education, delivered at an individual level, often fails to affect health outcomes, particularly over the long term. Recent results of successful trials addressing child obesity—studies which served as models for the design of our intervention—consistently show a failure in long-term impact [92,93,94,95,96]. The original plan for the CHALO! trial was to broaden the usual individual focus to include family members at the 8- and 14-month visits. However, engaging family members proved difficult. Husbands and fathers in the community worked long hours and were often unavailable. In some cases, the mother seemed unwilling to ‘share’ her private session with another family member. For these reasons, the family component was eventually dropped. Had we been able to succeed with this strategy, we might have had more success with caries and weight outcomes.

Mothers’ social networks likely play an important role in influencing health-related behaviors via multiple pathways, including social support, instrumental support, modeling, and social pressure/norms. Qualitative data collected during the study found that the influence of elders, predominance of family members in existing networks, along with low self-esteem and empowerment among mothers, was an obstacle in health behavior change [29]. It is challenging to engage mothers’ social networks in health behavior change. SA mothers are strongly influenced by the views of their elders, and express concern about their criticism [29]. Moreover, many SA mothers have sparse friendship networks, providing few sources of outside information and social support [97].

One solution to this problem is to construct an intentional social network—building new networks of participants from the ground up. This strategy, though complex, may be effective when ‘natural’, existing networks offer inadequate support or exert negative influences, such as disinformation, advocacy of unhealthy norms, and coercion [98,99]. The research team is currently engaged in the early phases of such a randomized control trial called the ‘Mothers’ Action for Child Health’ study, which aims to build intentional social networks among geographically proximally close SA mothers to increase social support and ultimately effect long-term changes to health-related behaviors. It may be that this more socially/contextually oriented approach will have better success than the traditional approach used in the CHALO! Study. A direction for future research is to seek to influence maternal social networks in order to support more robust changes, especially in the clinical outcomes of caries and obesity risk.

## 5. Conclusions

Results of the CHALO! study indicate improvements in our behavioral primary outcome—bottle/sippy cup use—and in most behavioral secondary outcomes, including dietary variables, maternal feeding practices, oral hygiene practices, and a large increase in dental visits, in the intervention group compared to controls. There was little evidence of change in the clinical outcomes of visible oral caries, though this outcome may have been influenced by measurement problems, and no change in weight gain velocity, a common obesity risk indicator. Social context and social constraints may have affected mothers’ ability to change their behavior enough to affect the clinical outcomes.

## Figures and Tables

**Table 1:** Study outcome variables, measures and constructs.

Outcome	Variable Type	Measures	Construct
Demographics	Sample characteristics	Demographics Questionnaire	Demographic data
Frequency of (Combined) Sippy Cup and Bottles	Primary outcome	Food Frequency Questionnaire (FFQ) [68]	Infant 7-day food inventory
Frequency servings of Sweeteners/Solid bottle additives	Secondary outcome	Food Frequency Questionnaire (FFQ) [68]	Infant 7-day food inventory
Frequency servings of Fruits and Vegetables	Secondary outcome	Food Frequency Questionnaire (FFQ) [68]	Infant 7-day food inventory
Frequency servings of Juice and Sweet Drinks	Secondary outcome	Food Frequency Questionnaire (FFQ) [68]	Infant 7-day food inventory
Frequency of Bottles/Sippy Cups (combined) at Nap or Bedtime	Secondary outcome	Food Frequency Questionnaire (FFQ) [68]	Infant 7-day food inventory
Frequency servings of Sweet and Salty Snacks	Secondary outcome	Food Frequency Questionnaire (FFQ) [68]	Infant 7-day food inventory
Frequency of Toothbrushing	Secondary outcome	Oral Hygiene and Dental Utilization Questionnaire [69]	Knowledge, self-efficacy of child oral health
Number of Dental Visits	Secondary outcome	Oral Hygiene and Dental Utilization Questionnaire	Knowledge, self-efficacy of child oral health
Feeding style	Secondary outcome, Mediator	Infant Feeding Styles Questionnaire (IFSQ) [70]	Infant Feeding Style
Physical Activity	Secondary outcome	Early Years Physical Activity Questionnaire (EYPAQ) [71]	Physical activity levels
Screen Time	Secondary outcome	Screen Time Questionnaire	Use of TV, computers, mobile phones
Visible Caries	Secondary outcome	Intra-oral camera	Oral health
Caries Severity	Secondary outcome	Intra-oral camera	Oral health
Weight gain velocity	Secondary outcome	Pediatrics visit	Obesity risk/speed of weight gain
Working Alliance between CHW and participants	Moderator	Working Alliance Inventory (WAI) [72]	Relationship warmth and rapport

**Table 2:** Structure of a typical session.

Activity	Goal	Time
Informal talk with Mom	Build rapport, provide support	10 min
Mom’s checklist and discussion	Assess progress, challenges, trouble shoot	10 min
Provide age-appropriate messages	Education, behavior change	10 min
Demonstrate healthy feeding/baby care	Skill building	10 min
Goal setting	Assess motivation, create an action plan	10 min
Summarize and check in	Build rapport	5 min

**Table 3:** Demographic characteristics for intervention and control groups.

Variable	Level	Overall N = 350	Control N = 174	Intervention N = 176
Child gender	Male	181 (52.62%)	86 (50.00%)	95 (55.23%)
	Female	163 (47.38%)	86 (50.00%)	77 (44.77%)
	N (Missing)	6	2	4
Number of children	1	134 (38.29%)	69 (39.66%)	65 (36.93%)
	2	150 (42.86%)	77 (44.25%)	73 (41.48%)
	3	61 (17.43%)	25 (14.37%)	36 (20.45%)
	4	3 (0.86%)	2 (1.15%)	1 (0.57%)
	5	2 (0.57%)	1 (0.57%)	1 (0.57%)
Years Mom in the US		5.93 (4.74)	6.34 (4.96)	5.53 (4.49)
Years of education (Mom)		13.54 (2.68)	13.67 (2.68)	13.42 (2.69)
Mom’s degree	Less than High School	57 (16.38%)	26 (15.03%)	31 (17.71%)
	High School Diploma	127 (36.49%)	59 (34.10%)	68 (38.86%)
	Some College	14 (4.02%)	9 (5.20%)	5 (2.86%)
	Associate’s	22 (6.32%)	10 (5.78%)	12 (6.86%)
	Bachelor’s	92 (26.44%)	53 (30.64%)	39 (22.29%)
	Advanced Degree	36 (10.34%)	16 (9.25%)	20 (11.43%)
	N (Missing)	2	1	1
Home ownership	Owned	58 (16.57%)	28 (16.09%)	30 (17.05%)
	Rented	283 (80.86%)	141 (81.03%)	142 (80.68%)
	Other arrangement	9 (2.57%)	5 (2.87%)	4 (2.27%)
Income	Under $19,999	46 (15.65%)	27 (17.76%)	19 (13.38%)
	$20,000–$39,999	156 (53.06%)	79 (51.97%)	77 (54.23%)
	$40,000–$59,999	42 (14.29%)	25 (16.45%)	17 (11.97%)
	$50,000–$59,999	50 (17.01%)	21 (13.82%)	29 (20.42%)
	N (Missing)	56	22	34

**Table 4:** Results of primary and secondary outcome measures for intervention and control group.

Outcomes Measured Longitudinally	Control				Intervention				*p*-Value Comparing Rate Ratios
	T0	T1	T2	Yearly Rate Ratio (95% CI)	T0	T1	T2	Yearly Rate Ratio (95% CI)	
Combined bottle/sippy cup use, median (IQR)	28.5 (0, 56)	21 (0, 35)	14 (0, 28)	0.58 (0.56–0.61)	28 (0, 49)	21 (0, 28)	7 (0, 21) $	0.36 (0.34–0.39)	<0.0001
Weekly bottle additives (IQR)	0 (0, 0)	0 (0, 1)	0 (0, 4)	9.31 (7.38–11.75)	0 (0, 0)	0 (0, 0) $	0 (0, 0.5) $$	3.39 (2.48–4.64)	<0.0001
Weekly frequency of fruits and vegetables, median (IQR)	7 (0, 14)	14 (14, 21)	14 (14, 21)	1.94 (1.82–2.06)	7 (0, 14)	21 (14, 28) $	21 (14, 21) $	2.30 (2.16–2.46)	0.0002
Weekly frequency of fruit juice, median (IQR)	0 (0, 0)	0 (0, 3)	2 (0, 7)	4.66 (3.87–5.61)	0 (0, 0)	0 (0, 1) $$$	0 (0, 2) $$$	2.45 (1.92–3.12)	<0.0001
Weekly frequency of sugary drinks, median (IQR)	0 (0, 0)	0 (0, 0)	0 (0, 0)	6.54 (4.11–10.40)	0 (0, 0)	0 (0, 0) $	0 (0, 0)	11.60 (4.26–31.58)	0.3
Nap/bedtime weekly quantity of bottle and/or sippy cup use, median (IQR)	0 (0, 2)	0 (0, 2)	0 (0, 2)	0.62 (0.49–0.79)	0 (0, 2)	0 (0, 1) $	0 (0, 0) $$$	0.23 (0.17–0.33)	<0.0001
Weekly frequency of teeth brushing, median (IQR)	0 (0, 4)	7 (3, 8)	7 (7, 14)	2.51 (2.23–2.83)	0 (0, 4)	7 (7, 10) $	10 (7, 14) $$	3.03 (2.72–3.37)	0.0226
	**T0**	**T1**	**T2**	**Yearly OR (95% CI)**	**T0**	**T1**	**T2**	**Yearly OR (95% CI)**	***p*** **-Value**
Weekly frequency of sweet and salty snacks, median (IQR) *	0 (0, 0)	4 (1, 10)	6 (2, 14)	40.68 (22.90–72.24)	0 (0, 0)	1 (0, 4) $$$	3 (0, 7) $$$	19.90 (11.11–35.67)	0.0543
Weekly frequency of unhealthy food, median (IQR) *	0 (0, 0)	7 (1, 14)	10 (5, 17)	50.62 (28.48–89.96)	0 (0, 1)	3 (0, 7) $$$	4 (1, 9) $$$	16.83 (9.67–29.3)	0.0023
	**T0**	**T1**	**T2**	**Yearly Increase (95% CI)**	**T0**	**T1**	**T2**	**Yearly Increase (95% CI)**	***p*** **-Value**
Velocity of weight gain, mean (SD) *****	17.4 (2.6)	21.6 (3.4)	25.3 (3.9)	7.10 (6.7, 7.47)	16.9 (2.4)	21.2 (2.8)	24.6 (3.4)	7.24 (6.86, 7.62)	0.6026
**Outcomes measured at T2**			**T2**				**T2**		***p*** **-Value**
Screen time, % **									0.0244
No screen time			3.85%				10.74%		
Less than 1 h/day			48.08%				52.35%		
1–4 h/day			42.95%				32.21%		
More than 4 h/day			5.13%				4.70%		
Dental visits, % ***			3.80%				37.25%		<0.0001
Time spent performing moderate/vigorous physical activity, mean (SD) ****			1728.21 (723.3)				1940.0 (925.0)		0.8062
Time spent on sedentary activity, mean (SD) ****			1029.5 (784.7)				841.4 (535.4)		0.4375
Frequency of caries incidence, % ***			3.13%				9.09%		0.2740

* For sweet and salty snacks and unhealthy food, proportional odds models with random effects were used. Yearly odds ratios for consumption of more sweet and salty snacks or unhealthy food, as well as their 95% CIs, are shown. ** Proportional odds model was used. *** Logistic regression model was used. **** Linear regression models were used. ***** For velocity of weight gain, we looked at monthly weight change and not yearly rate ratio. $ < 0.05; $$ < 0.001; $$$ < 0.0001.

**Table 5:** Mediation results.

RR of Bottle/Sippy Servings	Without Mediator	With Mediator	% Due to Mediator
T1	0.61	0.62	<1%
T2	0.49	0.49	<1%

## Data Availability

The data presented in this study will be made available by the authors on request.

## Supplementary Materials

The following supporting information can be downloaded at: https://www.mdpi.com/article/10.3390/ijerph23070837/s1, File S1. CHALO! consort_2025_checklist; Supplementary Table S1: Sample sizes for analysis across different timepoints; Supplementary Table S2: Baseline comparison of intervention vs. control groups of outcome variables; Supplementary Table S3: Multiple Imputation. Refs. [100,101] were cited in the Supplementary Materials File S1.
