# Supplementary material for: The CHALO! Study Results of a Randomized Controlled Trial to Reduce Risk of Childhood Dental Caries and Obesity
Source: Int J Environ Res Public Health. 2026 Jun 25;23(7):837. doi: 10.3390/ijerph23070837 (PMC13411643; doi:10.3390/ijerph23070837)
Supplement: Supplementary file 1 [file ijerph-23-00837-s001.zip › Chalo! supplementary table S1.pdf]

Supplementary Table S1: Sample sizes for analysis across different timepoints

Outcome variables	N		
	**T0**	**T1**	**T2**
Combined bottle/sippy	350	335	317
Weekly bottle additives	350	335	317
Weekly frequency of Fruits and Vegetables	350	335	317
Weekly frequency of Fruit juice	350	335	317
Weekly frequency of sugary drinks	350	335	317
Nap/bedtime weekly quantity of bottle and/or sippy cup use	350	334	311
Weekly frequency of sweet and salty snacks	350	335	317
Weekly frequency of unhealthy food	350	335	317
Weekly frequency of teeth brushing	350	173	266
