# Supplementary material for: The CHALO! Study Results of a Randomized Controlled Trial to Reduce Risk of Childhood Dental Caries and Obesity
Source: Int J Environ Res Public Health. 2026 Jun 25;23(7):837. doi: 10.3390/ijerph23070837 (PMC13411643; doi:10.3390/ijerph23070837)
Supplement: Supplementary file 1 [file ijerph-23-00837-s001.zip › Chalo! supplementary table S2.pdf]

Supplementary Table S2: Baseline comparison of intervention vs control groups of outcome variables

Variable	Overall N=350	Control N=174	Intervention N=176	P-Value
Weekly quantity of combined sippy cups and bottles	28 (0, 52)	28.5 (0, 56)	28 (0, 49)	0.445
Weekly servings of additives	0 (0, 0)	0 (0, 0)	0 (0, 0)	0.080
Weekly servings of fruits and vegetables	7 (0, 14)	7 (0, 14)	7 (0, 14)	0.353
Weekly servings of fruit juice	0 (0, 0)	0 (0, 0)	0 (0, 0)	0.188
Weekly servings of sugary drinks	0 (0, 0)	0 (0, 0)	0 (0, 0)	0.994
Frequency of child drinking from a bottle or sippy cup/day when put down to bed	0 (0, 2)	0 (0, 2)	0 (0, 2)	0.927
Weekly servings of sweets and salty snacks	0 (0, 0)	0 (0, 0)	0 (0, 0)	0.962
Weekly frequency of teeth cleaning	0 (0, 4)	0 (0, 4)	0 (0, 4)	0.900
Weekly servings of unhealthy food	0 (0, 1)	0 (0, 0)	0 (0, 1)	0.485
